# Estimation of surface doses in the presence of an air gap under a bolus for a 6 MV clinical photon beam - a phantom study

**DOI:** 10.1007/s00411-025-01106-6

**Published:** 2025-01-28

**Authors:** Dilson Lobo, Challapalli Srinivas, Sourjya Banerjee, M.S. Athiyamaan, K. Johan Sunny, Abhishek Krishna

**Affiliations:** Department of Radiation Oncology, Kasturba Medical College Mangalore, Manipal Academy of Higher Education, Manipal, India

**Keywords:** Air gap, Bolus, Surface dose, Radiotherapy

## Abstract

Goal of the present study was to develop and build a phantom that replicates the air gaps under a gel bolus and to estimate the surface dose (D_surf_) under normal incidence with a 6 MV photon beam. For this, an acrylic phantom with 10 plates, each including five open slots (one in the centre and four off axis) with a size of 2 cm × 2 cm at depths of 0.54 cm, 0.72 cm, 0.90 cm, 1.26 cm, and 1.62 cm from the phantom’s surface was used. Computed tomography image sets were obtained without and with a gel bolus (thickness: 2 mm, 4 mm, and 6 mm) placed on top of the phantom. Dose calculations were performed with the XiO treatment planning system (TPS) for a 6 MV photon beam at normal incidence and a field size of 15 cm × 15 cm that covered all the slots. A virtual bolus in TPS was employed in CT picture sets that did not include a bolus. Six points of interest at a depth of 1 mm from the surface contour of each slot were used to determine the mean surface dose (D_surf_) estimated by the TPS with and without the presence of a bolus. It turned out that, as the depth of the air gap (between skin surface and bolus surface) increased from 0.54 cm to 1.62 cm, there was a 25.2% increase in D_surf_ without bolus, followed by an increase of 7.6%, 6.4%, and 7.7% for a virtual bolus with 2 mm, 4 mm, and 6 mm thickness, while corresponding increases were 14.8%, 14.3%, and 8.3% for an actual bolus, respectively. However, as the thickness of the air gap increased, D_surf_ under the bolus decreased (from − 17.5% to -18.8%, and from − 10.4% to -16.9%, for a virtual and a physical bolus, respectively). It is concluded that, to ensure a homogeneous D_surf_ across the treatment area, extra attention should be given while utilizing a bolus in clinical radiation applications, to avoid any air gaps under the bolus.

## Introduction

Clinical radiotherapy (RT) treatments often include megavoltage photon beams, which typically result in a reduced tissue surface dose (D_Surf_) as compared to the peak dose (d_max_) at a certain depth in tissue. This phenomenon is known as “skin sparring” and it is estimated that D_Surf_ can be as low as 25% of the dose at d_max_. Therefore, when treating near-surface tumours, such as the scar region in post-mastectomy radiotherapy, dose adjustments are necessary to offset the skin-sparing effect. It is noted that D_Surf_ and d_max_ depend on photon beam energy, field size, beam modification devices, source to skin distance, and angle of incidence (Chao et al. 2011). To compensate for the skin sparring effect in such treatments, a layer of tissue-equivalent material called bolus is placed on the tissue surface, in order to increase D_Surf_. (Martin et al., 2000).

To prevent tumour recurrence in Postmastectomy Radiation Therapy (PMRT), it is essential that the chest wall surface receives exactly the prescribed dose. Bolus material is commonly used in PMRT, and in vivo dosimetry plays a critical role in 3D conformal radiotherapy (3D-CRT) and intensity-modulated radiotherapy (IMRT) for breast cancer treatment (Gul et al., 2024a). Due to the rigid contours of the human body, a bolus may not reach ideal contact with the patient, often resulting in air gaps between the bolus and the skin surface, which may disturb the D_Surf_. In fact, there are air gaps between the bolus and the chest wall because the bolus is usually free to move on the chest wall, and bolus location varies depending on the chest wall shape, bolus thickness, and material used. Several studies have been carried out to estimate the effect of an air gap between bolus and tissue surface on D_Surf_. Gul et al. conducted a study focusing on the impact of bolus use on skin dose in postmastectomy radiotherapy. Their research evaluated the effects using treatment planning system (TPS) calculations and measurements with thermoluminescence dosimeters (TLDs) across different techniques, including field-in-field IMRT, and helical tomotherapy (Gul et al., 2024b). Boman et al. investigated the dosimetric impact of D_Surf_ with 0.5 cm and 1.0 cm thick continuous air gaps below a bolus with a thickness of 0.5 cm, for left-side post-mastectomy radiotherapy in Volumetric Arc Therapy (VMAT), and standard practice in field treatments (Boman et al. 2018).

An air gap of 0.5 cm was shown to reduce head and neck surface dose by 9% (Mahdavi et al. 2015). For orthogonal static 6 MV beams with a field width of less than 10 cm ×10 cm, Khan et al. found a dose decrease of more than 10% at the surface for air gaps greater than 1.0 cm under the bolus, while for larger fields the dose reduction was negligible (Khan et al. 2013). According to Butson et al. a 1 cm air gap under the bolus can reduce the surface dose by up to 10% for 6 MV static beams, while the most significant dose decreases were found in lower field widths and angles that were closest to perpendicular direction of beam delivery (Martin et al., 2000). Gul et al. conducted a dosimetric evaluation to assess the impact of an air gap between tissue surface and bolus on dose distribution using parallel plate chambers (Gül et al., 2023). For 6 MV X-rays, the presence of an air gap was found to adversely impact the surface doses calculated by the TPS, demonstrating an inverse relationship between the air gap and surface dose.

By using a Markus parallel-plate chamber and a metal-oxide semiconductor field-effect transistor (MOSFET) dosimeter, Chung et al. investigated the effects of surface dose from air gaps with a thickness of up to 1 cm under bolus thicknesses of 0.5 cm and 1.0 cm in clinically used oblique photon beams (Chung et al., 2012). Accurate measurement of D_Surf_ doses in clinical radiotherapy treatments can provide valuable information to avoid near-surface tumour recurrence and at the same time to limit severe skin toxicity (Boman et al. 2018; Chung et al., 2012; Hsu et al. 2010). This all demonstrates that there is indeed a need to investigate the D_Surf_ in the presence of an air gap beneath the bolus. Consequently, the aim of the present study was to investigate D_Surf_ in the presence of an air gap beneath a gel bolus using a 6 MV clinical photon beam under normal incidence and a locally fabricated air gap slot phantom.

## Materials and methods

### Design and fabrication of an “air gap slot phantom”

**Fig. 1:**
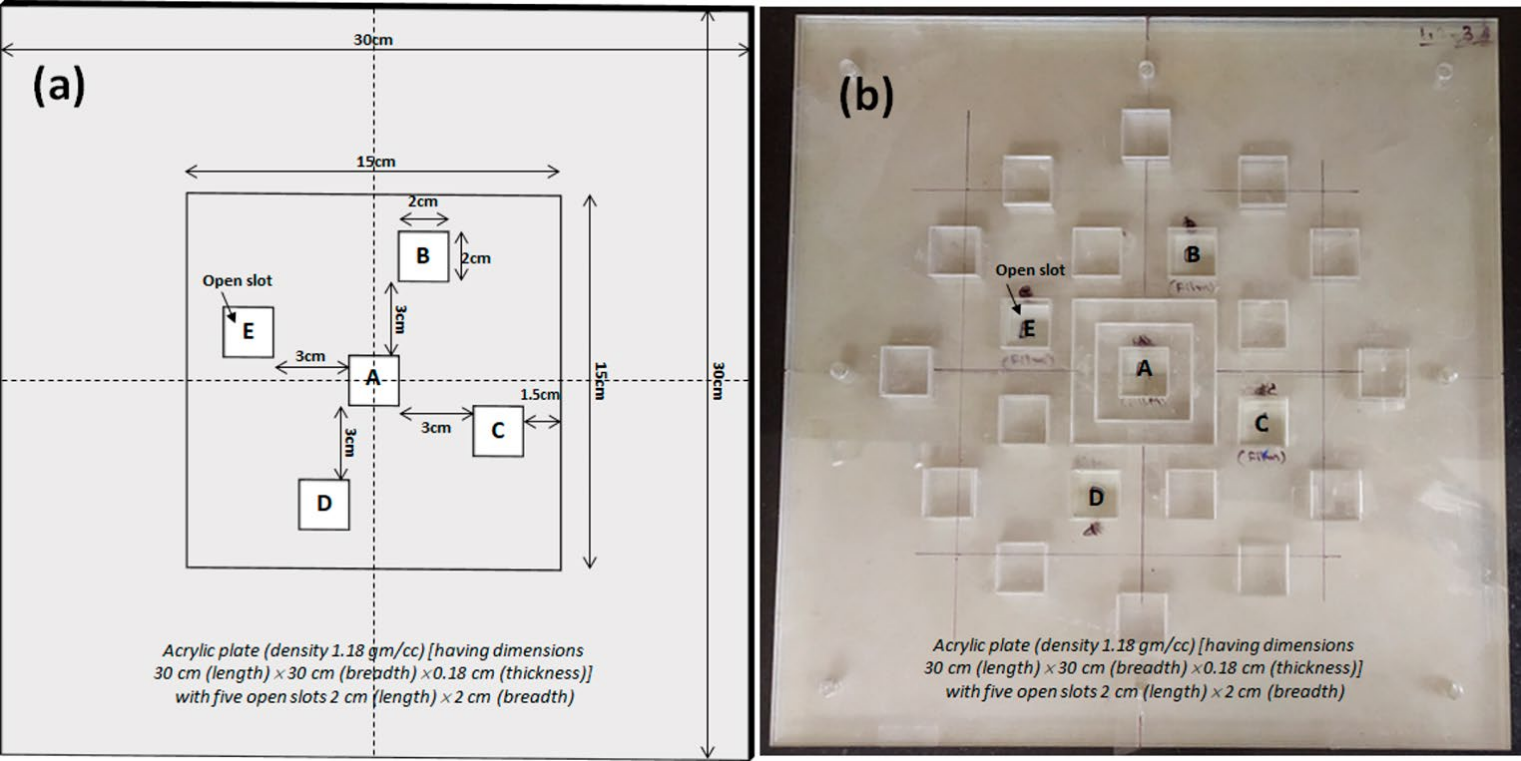
**a**) Schematic diagram of an acrylic plate including five open slots **A, B, C, D** and **E**) fabricated plate

An acrylic (ρ = 1.18 g/cm^3^) phantom including 10 plates, each measuring 30 cm (length) × 30 cm (width) × 0.18 cm (thickness) was supported by 13 solid water slabs (ρ = 1.045 g/cm^3^) each measuring 30 cm (length) × 30 cm (width) × 1 cm (thickness) to achieve full backscatter of photons was used. Five open slots (labelled as A, B, C, D, and E in Fig. 1) with dimensions of 2 cm × 2 cm were included in each plate in such a way that one was at the center and the remaining four were off-axis at a distance of 3 cm from the central slot. Figure 1a depicts a schematic diagram of an acrylic plate showing five open slots with dimensions, and distances of off-axis slots from the field edge. Initially there were multiple slots. However, provision was made in the phantom of this study and only five slots were considered and analyzed by filling the remaining slots, to make the phantom as homogenous as possible. Figure 1b shows the fabricated plate. The plate’s open slots can be closed as needed with an acrylic block of the same size as the slot.

**Fig. 2:**
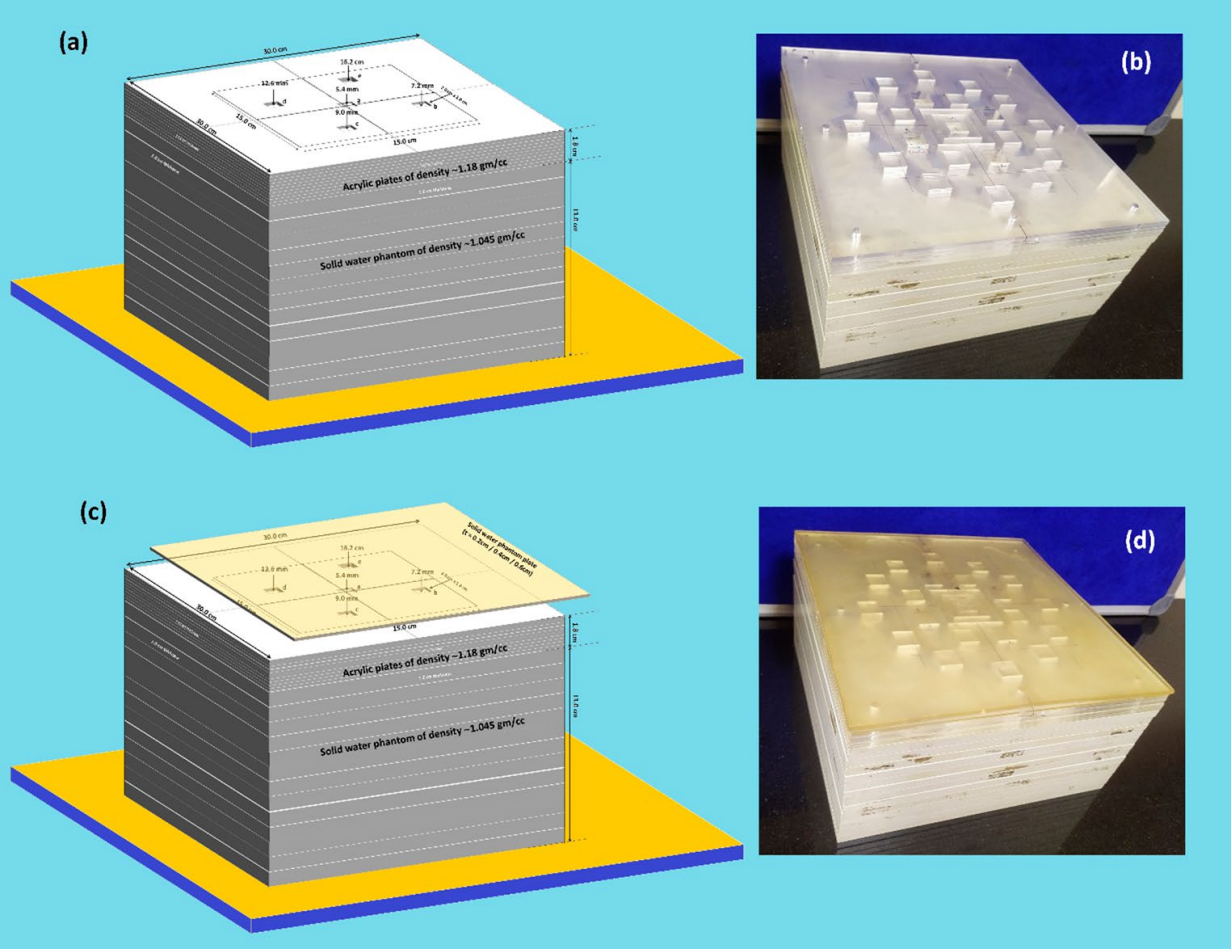
(**a**) schematic diagram (design) and (**b**) fabricated acrylic air gap slot phantom backed by solid water equivalent plates without bolus sheet on top of it (**c**) and (**d**) gel bolus sheet on top of the assembled phantom

The ten acrylic plates were assembled in such a way that the slots at A, B, C, D, and E were opened until the third, fourth, fifth seventh and ninth plate, respectively. As a result, the depths of each of the five slots beneath the bolus (when used) wre 0.54 cm, 0.72 cm, 0.90 cm, 1.26 cm, and 1.62 cm, respectively. In this way air gaps with different thicknesses beneath the bolus were created. Several super flab gel bolus (ρ = 1.02 g/cm^3^) sheets with dimensions 30 cm (length) × 30 cm (width) and thicknesses 2 mm / 4 mm / 6 mm were used on top of phantom. Figure 2a and b show the schematic diagram (design) and actually fabricated acrylic air gap slot phantom backed by solid water equivalent plates without bolus sheet on top of it, while Fig. 2c and d show the gel bolus sheet on top of the assembled phantom.

### Computed tomography (CT) simulation and contouring

The assembled phantom was scanned by means of a computerized tomography (CT) unit (GE LightSpeed (GE Medical Systems, Wisconsin, USA) with a 2.5-mm slice thickness, without and with a gel bolus sheet placed on top of it. The serial axial image data sets were exported to “Focalsim contouring station” (M/s Elekta Ltd., Crawley, UK) through Digital Imaging and Communications in Medicine (DICOM) network. Surface contour of the phantom was made and the contoured images were transferred to the treatment planning system (TPS).

### TPS dose estimation

The CMS XiO^®^ (Elekta Ltd, Crawly, UK) version 4.80.02 treatment planning system (TPS) was used for dose calculations using a superposition algorithm. To increase the dosimetric accuracy, a grid size of 2 mm was employed for TPS dose estimates.

### Surface dose estimates in presence of an air gap under gel bolus

Six interest points were placed 1 mm below the surface contour of each slot, to determine the surface dose in the absence / presence of a bolus. Beam placement (with 6 MV photon mode) was performed under normal incidence condition. A field size of 15 cm × 15 cm was used to cover all five slots, with the off-axis slots 1.5 cm away from the field size’s edge (Fig. 1a). Source-to-phantom surface distance (SSD) was maintained as 100 cm and 100 monitor units (MUs) were delivered. Dose calculated at 1.5 cm depth (d_max_ for 6 MV photon beam) was taken as 100%. The planning was carried out with all CT image sets. Aside from the CT image sets taken with gel bolus during the simulation process, a virtual bolus of thickness 2 mm, 4 mm, and 6 mm was used on top of the phantom in the TPS.

The dose calculated at interest points beneath each slot (without and with actual/virtual bolus) was noted, and the mean dose obtained from these interest points was referred to as the surface dose (D_surf_) measured beneath each slot in the absence / presence of a bolus in each plan was tabulated.

## Results

**Fig. 3:**
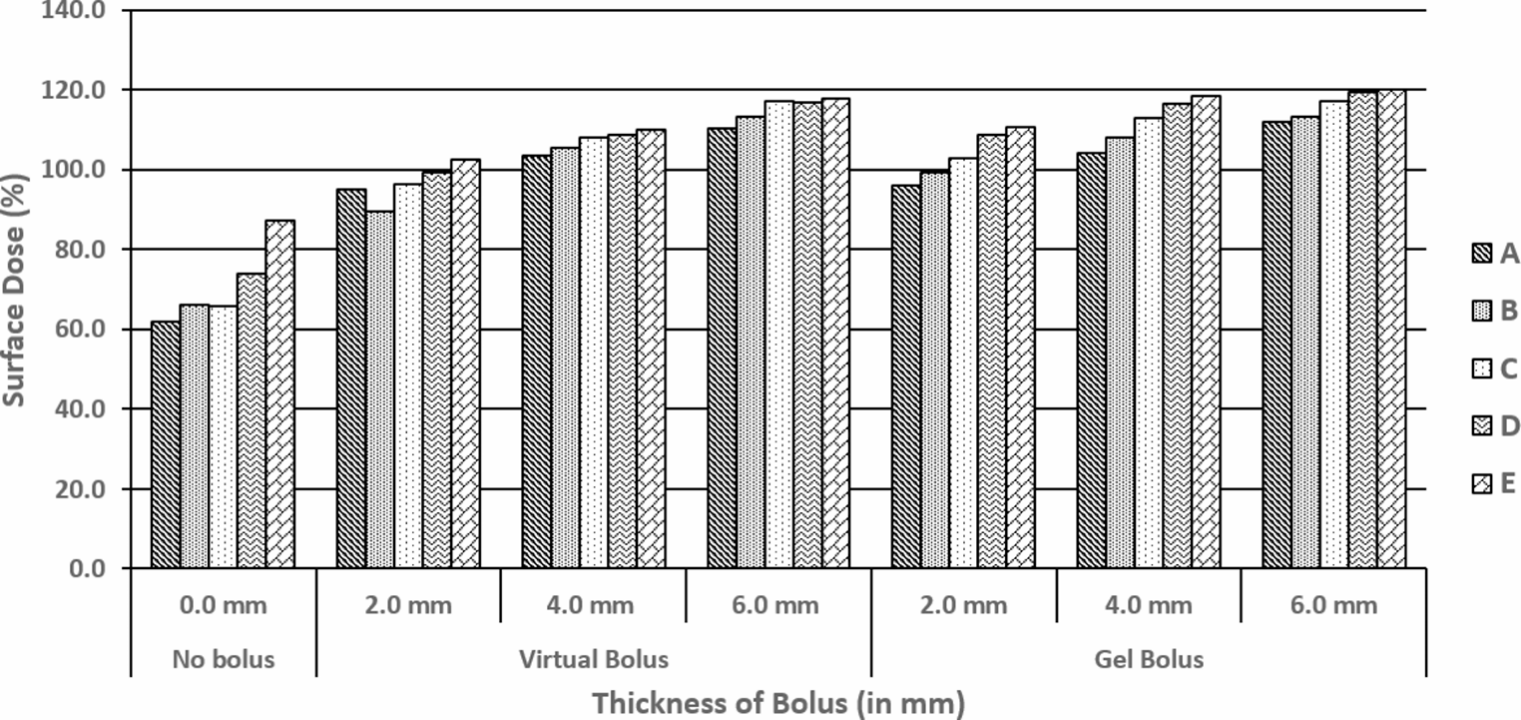
Treatment planning system (TPS) estimated surface dose (%) at five air gap slots (**A, B, C, D,** & **E**) with no bolus (0 mm thickness), with virtual and gel bolus of thickness of 2 mm, 4 mm, & 6 mm under normal incidence conditions of a 6 MV photon beam (using a 15 cm × 15 cm field size and source to phantom surface distance (SSD) of 100 cm)

Figure 3 shows the TPS estimated surface doses (%) at five air gap slots (A, B, C, D, & E) without (0 mm) and with gel bolus thickness of 2 mm, 4 mm, & 6 mm under normal incidence conditions of a 6 MV photon beam (using a 15 cm × 15 cm field size and an SSD of 100 cm). The graph depicts the data obtained from CT image sets in TPS with no bolus, virtual bolus, and actual bolus conditions.

As the depth of the air gap increased from 0.54 cm to 1.62 cm, there was a 25.2% increase in Dsurf without bolus, followed by an increase of 7.6%, 6.4%, and 7.7% for a virtual bolus with 2 mm, 4 mm, and 6 mm thickness, while corresponding increases were 14.8%, 14.3%, and 8.3% for an actual bolus, respectively.

The D_surf_ increases as the depth of the slot increases, both without and with the presence of a bolus (Fig. 3). Table 1 represents the D_surf_ (%) estimated by XiO TPS under the five air gap slots without and with application of gel bolus (virtual & actual) under normal incidence condition of a 6 MV photon beam (SSD = 100 cm and field size 15 cm × 15 cm). The table shows an increment / decrement of D_surf_ without and with bolus under each slot. This might probably be due to the lateral electron scatter contribution of the phantom in the slot.

**Table 1:** Dsurf (%) estimated by XiO TPS under air gap slots without and with presence of gel bolus (virtual & actual bolus application) under normal incidence condition with 6 MV photon beam (SSD = 100 cm and field size 15 cm x 15 cm)

Air gap Slot (depth in cm)	D_surf_ (%)												
	No bolus condition	Virtual Bolus condition						Actual Bolus condition					
	0.0 mm	2.0 mm	Increase	4.0 mm	Increase	6.0 mm	Increase	2.0 mm	Increase	4.0 mm	Increase	6.0 mm	Increase
A (0.54)	62.0	94.9	32.9	103.5	41.5	110.2	48.2	95.9	33.9	104.1	42.1	111.9	49.9
B (0.72)	66.0	89.5	23.5	105.3	39.3	113.1	47.1	99.4	33.4	108.0	42.0	113.3	47.3
C (0.90)	65.9	96.3	30.4	107.9	42.0	117.2	51.3	102.7	36.8	113.0	47.1	117.2	51.3
D (1.26)	73.9	99.3	25.4	108.7	34.8	116.8	42.9	108.6	34.7	116.6	42.7	119.3	45.4
E (1.62)	87.2	102.5	15.3	109.9	22.7	117.9	30.7	110.7	23.5	118.4	31.2	120.2	33.0
Increase	25.2	7.6	-17.6	6.4	-18.8	7.7	-17.5	14.8	-10.4	14.3	-10.9	8.3	-16.9

Table 1 illustrates that the surface dose increased at all air gap slots when bolus was applied. The surface dose increased as bolus thickness increased due to forward electrons from the bolus material that were generated by photon interaction. However, as the thickness of the air gap increased (from 0.54 cm to 1.62 cm), the surface dose under bolus application decreased (from − 17.5% to -18.8% and from − 10.4% to − 16.9% under virtual and actual bolus conditions, respectively).

## Discussion

In the presenthis study D_Surf_ was estimated in the presence of air gaps with different thicknesses of gel bolus in a locally fabricated air gap slot phantom with a 6 MV photon beam under normal incidence.

It was observed that increasing the air gap from 0.54 cm to 1.62 cm caused a notable rise in surface dose (D_surf_) without bolus, with a 25.2% increase. Smaller increases, ranging from 6.4 to 14.8%, were recorded with the application of a bolus. This indicates that D_surf_ rises with air gap depth, but the presence of bolus material (whether virtual or actual) helps to mitigate this increase compared to no bolus conditions. The increase in D_surf_ is likely due to lateral electron scatter from the phantom in the air gap.

It is emphasized that an accurate estimate of D_surf_ is critically important to prevent tumour recurrence and limit skin toxicity in radiotherapy. The results obtained in the present study align with those published in the literature, particularly regarding the role of air gaps in increasing surface dose and the mitigating effect of bolus application. Several studies support these findings, consistently showing that air gaps reduce surface dose, while bolus application helps to counteract this reduction.

Because a TPS calculation of D_Surf_ is somewhat inaccurate, a precise measurement of D_Surf_ can offer significant additional information in preventing tumour recurrence near the surface and limiting serious skin toxicity (Sakai et al. 2021; Robar et al. 2018). Specifically, skin dose measurements in air gaps under bolus may provide valuable information, in particular when irregular body surfaces are involved. Many studies have already reported the effects of air gaps under a bolus on surface dose (Martin et al., 2000; Boman et al., 2018; Khan et al. 2013; Mahdavi et al., 2015).

Wong et al. (2020) found that the TPS-calculated mean surface dose increased from 65% without bolus to 95% with a 5 mm bolus, highlighting the significant role of bolus in increasing D_surf_. Similarly, studies by Butson et al. (2000) and Boman et al. (2018) demonstrated a decrease in surface dose of up to 13.6% due to air gaps beneath the bolus in PMRT patients. The current study’s findings, particularly the reduction in D_surf_ with non-uniform air gaps, are consistent with these their results.

Gul et al. investigated the influence of air gaps between the surface and a 5 mm bolus on dose distribution, using TPS point doses. Their findings for 6 MV X-rays indicated that air gaps reduced TPS surface doses. Specifically, they identified a clear inverse relationship between the size of the air gap and the surface dose, demonstrating that larger air gaps led to lower surface dose levels.

Fiedler DA et al. (Fiedler et al. 2021) reported that from the planned tangential field in field 3DCRT techniques, the TPS-estimated and radiochromic-film-measured percentage surface doses obtained from a chest phantom without bolus, and with a 5 mm and 10 mm bolus were 60.6 ± 11.7% and 63.7 ± 3.0%; 97.2 ± 1.1% and 96.8 ± 2.8%; and 96.4 ± 1.7% and 94.4 ± 3.5%, respectively.

Ordonez-Sanz C et al. (Ordonez-Sanz et al. 2014) reported that the TPS-calculated and TLD-measured percentage surface doses under tangential irradiation technique without bolus application were 76.5% and 68.0% respectively. D_Surf_ values obtained in the present study (Table 1) for no bolus, a 5 mm, and a 10 mm bolus are in good agreement with the above-mentioned published results.

Butson et al.(Martin et al., 2000) reported that a 10 mm air gap under the bolus can reduce the surface dose by up to 10% for 6 MV static beams using parallel plate chamber with small-sized and/or 60^0^ angled fields. Boman et al. (Boman et al. 2018) explored the surface dose with uniform air gap thicknesses of 5 mm & 10 mm below a 5 mm thick gel bolus in Volumetric Intensity Modulated Arc Therapy (VMAT) as well standard practice in field techniques in PMRT patients. They observed that an air gap of 10 mm under a bolus in VMAT plans reduced the surface dose up to 13.6%, which would result in a clinical impact on tumour recurrence rate. Mahdavi et al. (Madhavi et al., 2015) reported that an air gap of 5 mm thickness under a 5 mm thick gel bolus reduced the surface dose by 9% in head and neck treatments as observed from a treatment planning system (TPS).

Khan et al. (Khan et al. 2013) demonstrated that, for a 6 MV photon beam, reduction of surface dose (by about 10%) becomes significant for air gaps > 5 mm below a gel bolus during IMRT therapy plans. By using a Markus parallel-plate chamber and a metal-oxide semiconductor field-effect transistor (MOSFET) dosimeter, Jin-Beom Chung et al. (Chung et al., 2012) investigated the effects on surface dose from air gaps of up to 10 mm under 5 mm and 10 mm thick boluses in oblique 6 MV photon beams with a solid water phantom. With 60^0^ angle incidence, they observed an about 9.2% and 10.3% reduction of surface dose with a 10 mm air gap under 5 mm and 10 mm bolus applications, respectively.

Fiedler et al. (2021) conducted a study to compare skin doses for PMRT using tomotherapy and Halcyon, revealing that TLD doses were higher than treatment planning system (TPS) doses, with tomotherapy showing higher skin doses than Halcyon.

All the above studies addressed a D_surf_ reduction in various treatment techniques in the presence of uniform air gaps under a gel bolus. The values for D_surf_ obtained in the present study at different thicknesses of bolus applications with air gaps agree with the above-mentioned published results. However, for non-uniform air gaps, the surface dose reduction was minimal (3 to 3.5%) compared to the presence of uniform air gaps. So-Yeon Park et al. (Part et al., 2016) compared a 3D-printed bolus and a commercial bolus in PMRT. They observed average surface dose differences of -3.2% and − 1.1%, for a 5 mm commercial bolus and a 5 mm 3D-printed bolus (made of polylactic acid), respectively, indicating that the 3D-printed bolus did not only reduce the daily positioning error, but also overcome the dose reduction caused by the air gap between bolus and skin surface.

## Conclusion

Based on the findings of the present study it is concluded that special precautions must be taken to avoid unwanted air gaps beneath a bolus, in order to avoid any unwanted decrease in surface dose and to achieve a uniform surface dose across the treatment region. It is noted, however, that a customized 3D-printed bolus may be a better option as quoted in the literature rather than a commercially available other bolus. Further research should be conducted to investigate the effect of air gaps on D_surf_ at different bolus thicknesses for irradiation conditions other than normal incidence, using different photon energies and field sizes. Performing dose measurements with various dosimeters would also be useful to validate TPS-calculated surface doses experimentally.

## Data Availability

No datasets were generated or analysed during the current study.
